# Predominant Rab-GTPase amplicons contributing to oral squamous cell carcinoma progression to metastasis

**DOI:** 10.18632/oncotarget.4277

**Published:** 2015-06-15

**Authors:** Sabrina Daniela da Silva, Fabio Albuquerque Marchi, Bin Xu, Krikor Bijian, Faisal Alobaid, Alex Mlynarek, Silvia Regina Rogatto, Michael Hier, Luiz Paulo Kowalski, Moulay A. Alaoui-Jamali

**Affiliations:** ^1^ Department of Otolaryngology Head and Neck Surgery, Sir Mortimer B. Davis-Jewish General Hospital, Canada; ^2^ Segal Cancer Centre and Lady Davis Institute for Medical Research, Sir Mortimer B. Davis-Jewish General Hospital, Departments of Medicine, Oncology, and Pharmacology and Therapeutics, Faculty of Medicine, McGill University, Canada; ^3^ Department of Head and Neck Surgery and Otorhinolaryngology, AC Camargo Cancer Center and National Institute of Science and Technology on Oncogenomics (INCITO), Brazil; ^4^ NeoGene Laboratory, Department of Urology, Faculty of Medicine, UNESP, and International Research Center (CIPE), AC Camargo Cancer Center, Brazil; ^5^ Inter-institutional Grad Program on Bioinformatics, University of São Paulo, Brazil

**Keywords:** aCGH, genomic, metastasis, oral squamous cell carcinoma, rab GTPases

## Abstract

Metastatic oral squamous cell carcinoma (OSCC) is frequently associated with recurrent gene abnormalities at specific chromosomal *loci*. Here, we utilized array comparative genomic hybridization and genome-wide screening of metastatic and non-metastatic tongue tumors to investigate genes potentially contributing to OSCC progression to metastasis. We identified predominant amplifications of chromosomal regions that encompass the *RAB5*, *RAB7* and *RAB11* genes (3p24-p22, 3q21.3 and 8p11–12, respectively) in metastatic OSCC. The expression of these Rab GTPases was confirmed by immunohistochemistry in OSCC tissues from a cohort of patients with a follow-up of 10 years. A significant overexpression of Rab5, Rab7 and Rab11 was observed in advanced OSCC cases and co-overexpression of these Rabs was predictive of poor survival (log-rank test, *P* = 0.006). We generated a Rab interaction network and identified central Rab interactions of relevance to metastasis signaling, including focal adhesion proteins. In preclinical models, mRNA and protein expression levels of these Rab members were elevated in a panel of invasive OSCC cell lines, and their down-regulation prevented cell invasion at least in part via inhibition of focal adhesion disassembly. In summary, our results provide insights into the cooperative role of Rab gene amplifications in OSCC progression and support their potential utility as prognostic markers and therapeutic approach for advanced OSCC.

## INTRODUCTION

Oral squamous cell carcinoma (OSCC) is the most common subtype of malignant tumors of the head and neck with an incidence ranked as the eighth most frequent cancer worldwide [1]. Annually, it is estimated that 145,500 deaths are caused by OSCC, of which 96,720 occur in developing countries [2]. Advances in surgical and therapeutic modalities for OSCC management have led to a substantial improvement in survival rates. However, the overall 5-year survival remains among the lowest compared to common cancers, particularly for metastatic OSCC where currently available therapeutic modalities have a limited efficacy [3, 4]. Indeed, distant metastasis and high incidence of recurrence are the primary contributors of OSCC-related mortality [5–7].

Aggressive forms of OSCC are associated with recurrent chromosomal aberrations. The magnitude and frequency of these chromosomal alterations and their impact on gene transcription have been investigated at the genomic scale [8–11]. Of relevance to this study, genes involved in the regulation of intracellular vesicular trafficking, including endocytosis, have been demonstrated to control key signaling steps required for the metastatic process [12, 13]. In particular, deregulated expression of Rab GTPase family members have been observed in advanced cancers, where in many instances Rab overexpression has been implicated in the regulation of autocrine and paracrine cell signaling that control metastasis development [14–18].

Rabs belong to the Ras superfamily of small-GTPases, which in mammalian cells includes over 70 members [19]. These proteins cycle between GDP- and GTP-bound forms via nucleotide exchange catalyzed by a GDP/GTP exchange factor. In their inactive GDP-bound form, Rabs interact with few partners such as escort proteins and GDP dissociation inhibitor. In their GTP-bound form, Rabs recruit a wide range of effector proteins to regulate both endocytic and secretory pathways involved in vesicle budding, motility, tethering, fusion and membrane fission; these steps are crucial for the process of vesicular transport and protein trafficking [19, 20]. Moreover, Rabs regulate additional mechanisms including the formation of exosomes, which are involved in the regulation of tumor microenvironment and metastasis homing [21–25].

In the present study, we carried out a high-resolution array comparative genomic hybridization (aCGH) analysis and genome-wide screening on laser capture microdissected surgical specimens from a cohort of patients with non-metastatic and metastatic OSCC. We identified chromosomal amplicons corresponding to members of the Rab GTPase family, namely Rab5, Rab7 and Rab11, to be associated with OSCC progression to metastasis. Co-amplification and co-expression of these multiple Rabs were significantly increased in advanced OSCC and were predictive of poor overall survival. In preclinical OSCC models, these Rab members were found to greatly impact OSCC cell locomotion and invasion, at least in part via regulation of focal adhesion turnover.

## MATERIALS AND METHODS

### Study population

This study was approved by the Research Ethics Committees of AC Camargo Cancer Center (Brazil) and Jewish General Hospital (Canada). Patients were advised of the procedures and provided written informed consent. Ethical guidelines were followed and samples and clinicopathological data were handled in a coded fashion. Eligibility criteria included previously untreated patients, without a second primary tumor and submitted for treatment in the same institution.

Twenty OSCC samples were removed surgically from the tongue of patients who developed distant metastasis (10 cases) and patients without recurrence or evidence of metastatic disease (10 cases) during 157 months follow-up. Tissue sections (5 μm thick) were transferred onto glass slides, stained with hematoxylin and eosin (H&E) and assessed by a certified pathologist to identify appropriate tumor areas during the laser capture microdissection (LCM). These samples were used for aCGH experiments. For biomarker validation by immunohistochemistry, an independent set of 52 OSCC paraffin-embedded tissue specimens were collected from 12 patients who had recurrence or distant metastasis and 40 OSCC patients with no evidence of progression and good outcome after a follow-up for up to 120 months. Matched morphologically normal samples from the surgical margins were used as controls. For all cases tumor staging was reclassified according to the 2002 version of the International Union Against Cancer (TNM). Histological grade was determined on the basis of the classification by the World Health Organization [26]. The medical records were examined to obtain detailed characteristics of the studied patients (Table 1).

**Table 1:** Distribution of the OSCC cases according to demographic, lifestyle, and clinical variables

Variable	Category	Fresh samples *n* (%)		Paraffin-embedded samples *n* (%)
		Non-metastatic	Metastatic	
Age	< 50 year	5 (50)	2 (20)	8 (15.4)
	≥ 50 year	5 (50)	8 (80)	44 (84.6)
Gender	Male	7 (70)	10 (100)	31 (59.6)
	Female	3 (30)	0	21 (40.4)
Smoking habit	No	4 (40)	2 (20)	23 (44.2)
	Yes	6 (60)	8 (80)	29 (55.8)
Alcohol consumption	No	3 (30)	1 (10)	15 (28.8)
	Yes	7 (70)	9 (90)	37 (71.2)
Clinical stage	T1 + T2	9 (90)	4 (40)	32 (61.5)
	T3 + T4	1 (10)	6 (60)	20 (38.5)
Lymph nodes	N0	9 (90)	3 (30)	40 (76.9)
	N+	1 (10)	7 (70)	12 (23.1)
Recurrence or metastasis	No	10 (100)	0	40 (76.9)
	Yes	0	10 (100)	12 (23.1)
Status	Alive	10 (100)	6 (60)	45 (86.5)
	Died	0	4 (40)	7 (13.5)

### Laser capture microdissection (LCM) and DNA isolation

Genomic DNA was obtained from fresh tumor samples after LCM using the PixCell^®^ II Laser Capture Microdissection System (Arcturus Engineering, Mountain View, CA, USA). Briefly, genomic DNA was isolated from approximately 3,000 cells captured from 5 μm frozen sections mounted onto glass slides and stained with H&E using DNeasy Tissue Kit (Qiagen, Chatsworth, CA, USA). The quantification and quality of the DNA samples were evaluated using NanoDrop^®^ (ND-1000 Spectrophotometer v.3.0.1, Labtrade, Wilmington, NC, USA) and Bioanalyzer (Agilent Technologies, Palo Alto, CA, USA), respectively.

### Array comparative genomic hybridization (aCGH) and data analysis

Genomic DNA samples from OSCC and normal tissue (Promega, Madison, WI, USA) were differentially labeled using the Genomic DNA Enzymatic Labeling Kit (Agilent Technologies). The hybridizations were performed on Agilent Human CGH 44K Oligo Microarrays according to the manufacturer's recommendations. The aCGH images were acquired with a DNA microarray scanner using SureScan High-Resolution Technology and the Scan Control (version 8.1) software program (Agilent Technologies). Genomic Workbench software (Agilent Technologies) with the statistical algorithm ADM-2, and sensitivity threshold 6.0 was used to investigate chromosomal patterns within the microarray profiles. These parameters were used to define the following: copy number gain (≥ 0.6), copy number loss (≤ 0.8), and homozygous loss (≤ −1.2). Unsupervised clustering was utilized to identify the grouping profiles. Hierarchical clustering was performed using Euclidean distance and complete linkage with 1000 permutations. The molecular processes, functions and molecular networks were further evaluated by analyzing alterated genes using Ingenuity Pathways Analysis (IPA) (http://www.ingenuity.com). Protein-protein interaction (PPI) networks were annotated by the I2D database (http://ophid.utoronto.ca), visualized and analyzed using NAViGaTOR v2.03 (http://ophid.utoronto.ca/navigator/).

### Primers and quantitative real time RT-PCR (qRT-PCR)

cDNAs were synthesized from 1 μg of isolated RNA using Superscript II reverse transcriptase (Invitrogen, Carlsbad, CA, USA) and random primers (Invitrogen). Primer set sequences was chosen using the Primer Express 3.0 software (http://frodo.wi.mit.edu/cgi-bin/primer3/primer3_www.cgi) (Supplementary Table S1). qRT-PCR amplification was conducted in a total volume of 20 μL, using Power SYBR Green PCR Master Mix (Applied Biosystems, Foster City, CA, USA) and quality controls as proposed by MIQE Guidelines [27]. The reactions were carried out in triplicate. *GAPDH* was the most stable control gene from four endogenous genes tested (*GAPDH*, *ACTB*, *HPRT1* and *BCR*) using the geNorm algorithm [27]. Fold differences in the relative gene expression were calculated using Pfaffl model [28].

### Tissue microarray (TMA) platform

Tissue cores with a dimension of 1.0 mm from each specimen were punched and arrayed in duplicate on a recipient paraffin block using a Tissue Microarrayer (Beecher Instruments^®^, Silver Springs, MD, USA). After cutting sections from the recipient block and transferring these with adhesive tape to coated slides for subsequent UV cross-linkage (Instrumedics Inc^®^, Hackensack, NJ, USA), the slides were dipped in a layer of paraffin to prevent oxidation and stored in a freezer at −20°C.

### Immunohistochemistry (IHC) analysis and scoring

IHC was carried out on TMA as described earlier [29]. Incubations with the primary antibodies were conducted overnight at 4°C for: anti-Rab5 (1:100; Cell Signaling, Danvers, MA, USA), anti-Rab7 (1:100; Cell Signaling), and anti-Rab11 (1:50; Santa-Cruz, CA, USA). The sections were washed and incubated with secondary antibodies (Advanced ^™^ HRP Link, DakoCytomation, K0690, Denmark) followed by the polymer detection system (Advanced ^™^ HRP Link, DakoCytomation). Reactions were developed with a solution containing 0.6 mg/mL of 3,3′-diaminobenzidine tetrahydrochloride (DAB, Sigma, St Louis, MO, USA) and 0.01% H_2_O_2_ and then counter-stained with Mayer's hematoxylin. Positive controls (a tissue known to contain the antigen under study) were included in all reactions in accordance with manufacturer's protocols. The negative control consisted in omitting the primary antibody and incubating slides with PBS and replacing the primary antibody with normal serum. The IHC reactions were performed in duplicate on different TMA levels, representing four-fold redundancy for each case. The second slides were 25 sections deeper than the first, resulting in at least 250 μm of distance between the two sections with different cell samples for each tumor.

IHC analysis was performed blindly to the clinical aspects and conducted by two independent certified pathologists. Each core was scanned in low power field to choose the most stained area predominant in at least 10% of tumor cells [30]. The presence of a clearly visible dark brown precipitation was considered positive for immunostaining. Slides were analyzed as previously described [31] in accordance to the staining intensity: 0, no visible reaction; 1, weak expression, 2: strong positivity. For statistical analysis, the samples were categorized into groups: negative and positive cases.

### Statistical analysis

Statistical analyses of associations between variables were performed by the Fisher's exact test (with significance set for *P* < 0.05) and for continuous variables the non-parametric Mann–Whitney *U* test. Survival probabilities were analyzed by the Kaplan–Meier method. The log-rank test was applied to assess the significance of differences among actuarial survival curves with a 95% confidence interval. All analyses were performed using the statistical software package STATA-13 (STATA Corporation, College Station, TX, USA).

### Cell culture

The oral cancer cell lines SCC-9 and SCC-25 (ATCC, Manassas, VA, USA) were maintained in DMEM/F12 medium (Invitrogen) supplemented with 10% fetal bovine serum (FBS, Mediatech Inc, Herndon, VA, USA), 400 ng/mL hydrocortisone and 100 μg/mL gentamycin and kanamycin at 37°C in the humidified atmosphere of 5% CO_2_. OSCC1.2 cell line was established by this group from a metastatic OSCC and maintained in culture as described earlier [32, 33]. The normal oral epithelial (NOE) cells were isolated from normal human tongue tissue and maintained in culture in serum-free KSF medium supplemented with 5 μg/mL of bovine pituitary extract as described previously [34].

### siRNA expression

Knockdown of each of the targeted Rab GTPases was achieved using siRNA transfection. Target sequences used were: *RAB5* 5′-GCAAGCAAGUCCUAACAUU-3′, *RAB7* 5′-CTGCTGCGTTCTGGTATTTGA-3′, and *RAB11* 5′-GAGUAAUCUCCUGUCUCGA-3′. Transfections were carried out using 100 nM of siRNA oligonucleotides incubated with DharmaFECT1 (Thermo Fisher Scientific, Lafayette, CO, USA) in Opti-MEM I reduced serum medium (Invitrogen) according to the manufacturer's instructions.

### Western blot analysis

Total cell extracts were used for western blotting as described [35]. Blots were detected using the antibodies for anti-Rab5 (1:1000; Cell Signaling), anti-Rab7 (1:1000; Cell Signaling), anti-Rab11 (1:1000; BD Transduction, San Jose, CA, USA), and anti-GAPDH (1:10000; Cedarlane Lab, Hornby, ON, Canada). Staining signal was detected with peroxidase-conjugated secondary antibodies and enhanced chemiluminescence detection system.

### Invasion assay and migration assay

Cell invasion was quantified using 8 μm porous chambers coated with BD Matrigel Matrix (BD Biosciences, Bedford, MA, USA) according to the manufacturer's recommendations. Cell migration was assayed using the qualitative wound-healing assay. Each experiment was performed at least three times and results are expressed as average ± SD. Statistical significance was analyzed using the Student's *t* test.

### Live cell locomotion assay

Cells were seeded at low density on multi-well chambered coverglass (LabTek, Rochester, NY, USA). After starving, cells were stimulated with 10 ng/mL EGF and plated on a heated humidified stage supplied with 5% CO_2_. Phase contrast time-lapse images of an average of 30 cells per condition were captured every eight minutes for four hour by optimized Nipkow spinning disk confocal microscope (WaveFx spinning disk, Quorum Technologies Inc, Guelph, ON, Canada). Cell motility was measured by tracing the cell periphery manually using Volocity software (Perkin Elmer, Waltham, MA, USA). This time frame was determined based on a pilot study to determine the rate of focal adhesion disassembly in control cells.

### Live cell imaging of focal adhesion turnover

For fluorescence imaging of focal adhesions in single live cell, cells were transfected with GFP-paxillin and plated on a multi-well chambered coverglass (LabTek). After starving, cells were stimulated with 10 ng/mL EGF and placed on a heated humidified stage supplied with 5% CO_2_. Fluorescent images were captured every eight minutes for four hours using a heated 63x/1.40 NA objective at the optimized Nipkow Spinning Disk confocal microscope (WaveFx spinning disk, Quorum Technologies Inc). Cooled CCD camera control and image acquisition was done using Volocity imaging software (Perkin Elmer). At the extremely high speed, different cells were followed in intervals less than 10 seconds. Fluorescence intensities of individual adhesions from background-subtracted images were measured over time using Volocity imaging software, and quantified as described [36]. Measurements were made at least in 25 individual adhesions from 20 separate cells for each condition. Duration measurements were made for these same adhesions by counting the amount of time lapsed between the first and last frames in which an individual adhesion was observed.

## RESULTS

### RAB genes are clustered within the gained/amplified chromosomal regions in metastatic OSCC

**Figure 1:**
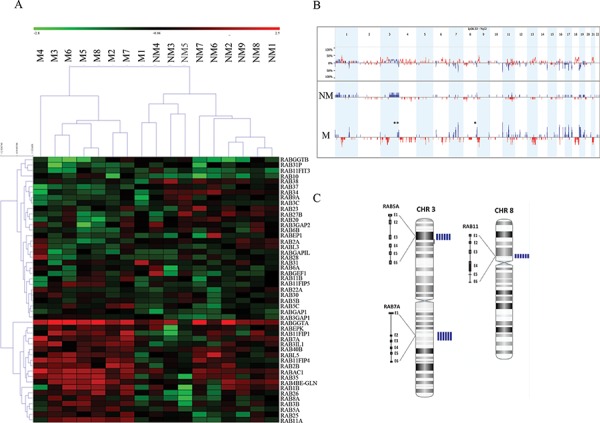
A. A representation of entire unsupervised cluster based on the copy number data of Rab genes according to clinical status (metastatic OSCC *versus* non-metastatic) We observed that metastatic OSCC samples (M4, M3, M6, M5, M8, M2 and M7) had similar copy number gains of specific members of the RAB gene family. **B.** The frequency plot of copy number gains (blue) and losses (red) identified a large number of chromosomal imbalances in more than 80% of OSCC invasive cases. The highest scoring *loci* identified in this analysis were mapped to regions of recurrent copy number gain in metastatic OSCC, including 3p24-p22, 3q21.3 and 8p11–12. These regions correspond to *RAB5*, *RAB7* and *RAB11* respectively. **C.** Array CGH profile in chromosomes 3 and 8 showing an increased copy numbers (amplification) in the sequences for *RAB5*, *RAB7* and *RAB11*. The blue vertical bars on the right to each chromosome represent the number of metastatic cases showing gained/amplified region per patient. M: metastatic group; NM: non-metastatic cases.

aCGH analysis combined with clinicopathological information from a cohort of patients with non-metastatic (*n* = 10) and metastatic (*n* = 10) OSCC with a follow-up of up to 157 months were exploited to infer Rab expression “footprints” of recurrent genomic amplicons in relation to risk of OSCC metastasis, recurrence and overall survival (Table 1). Analysis of the genomic data allowed the clustering of cases regardless of the clinical features, indicating that Rab markers are potentially relevant to OSCC progression. In particular, expression levels of *RABGGTA*, *RABEPK*, *RAB11FIP1*, *RAB7A*, *RAB3IL1*, *RAB40B*, *RABL5*, *RABA11FIP4*, *RAB2B*, *RABAC1*, *RAB35*, *RAB4BE-GLN*, *RAB1B*, *RAB26*, *RAB8A*, *RAB3B*, *RAB5A*, *RAB25* and *RAB11A* were able to segregate metastatic *versus* non-metastatic OSCC groups (Figure 1A). The frequency plots of copy number gains (blue) and losses (red) in metastatic *versus* non-metastatic OSCC identified chromosomal imbalances in more than 80% of invasive cases (Figure 1B). The prevalence of common genomic amplifications was seen in chromosomal regions that encompass several Rab GTPases. The highest scoring *loci* identified in this analysis were mapped to regions of recurrent copy number gain in aggressive OSCC, including 3p24-p22, 3q21.3 and 8p11–12, which correspond to *RAB5*, *RAB7* and *RAB11*, respectively (Figure 1C; Supplementary Tables S2–S4). Moreover, a cross-reference of the Rab gene clusters among a list of differentially expressed genes seen in OSCC, retrieved from the database collected and processed at the NCBI-GEO database (http://www.ncbi.nlm.nih.gov/geo/), which originated from ten studies (654 samples) reported in the literature, showed 72 genes to be differentially regulated in OSCC (*P* < 0.05; Supplementary Table S5). Amongst these 72 genes, 61 can be classified as Rab interacting proteins obtained from protein-protein interaction databases (MINT (mint.bio.uniroma2.it/mint/), HPRD (http://www.hprd.org) and IntAct IntAct (www.ebi.ac.uk/intac/)) (*P* < 0.05; Supplementary Table S6).

### Co-overexpression of Rab5, Rab7 and Rab11 is predictive of OSCC progression to metastasis and poor prognosis

**Figure 2:**
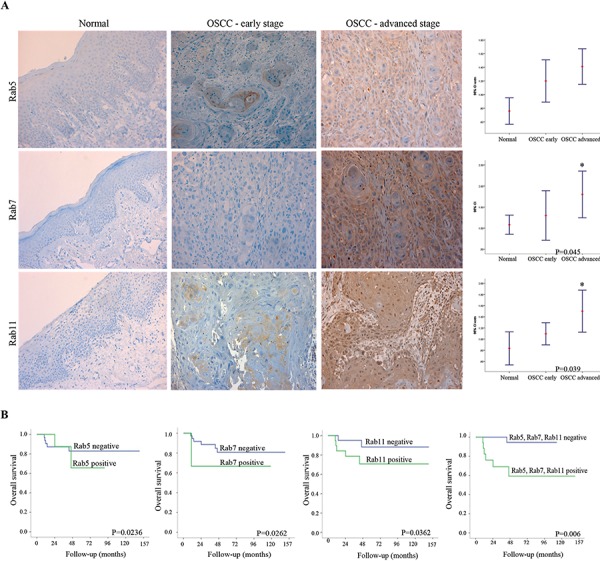
A. Representative images of immunostaining for Rab5, Rab7 and Rab11 proteins in normal (left side) and OSCC samples (right side) A weak staining was observed in non-cancer tissues while a moderate to strong cytoplasmic staining was detected in OSCC samples. Original magnification: 200X. Rab protein was differentially expressed in early and advanced clinical stages of OSCC patients as well as in the morphologically normal tissue. Confidence intervals (95%) show normalized mean intensity value units as determined by evaluation of immunohistochemistry. The Y axis represents numerical values corresponding to the intensity of expression. **B.** The survival probability, analyzed by Kaplan-Meier test, revealed a short survival rate for Rab5, Rab7 and Rab11 (log-rank test, *P* = 0.0236, *P* = 0.0262 and *P* = 0.0362 respectively) while co-overexpression of these Rab members revealed the strongest association with worst overall survival (log-rank test, *P* = 0.0006).

The expression levels of Rab5, Rab7 and Rab11 proteins were investigated in relation to the clinicopathological parameters using paraffin-embedded OSCC tissue specimens (Table 2). In agreement with the genomic data, overexpression of specific Rab proteins correlated with OSCC clinical features, in particular with advanced stages (Figure 2A; Rab7 and Rab11; *P* = 0.045 and *P* = 0.039 respectively) and tumor recurrence (Rab5, *P* = 0.050). High expression levels of Rabs were observed in invasive OSCC tissues (overall expression values of Rab5, Rab7 and Rab11 were 80.8%, 90.4%, and 55.8%, respectively), while in morphologically normal oral epithelium surrounding cancer lesions expression of these Rabs was very low to undetectable (Figure 2A). Statistical analysis revealed a positive correlation of Rab5 staining with the occurrence of tumor relapse (*P* = 0.050) (Table 2). There were no significant associations between overexpression of any of the identified Rab members and the type of treatment (Rab5: *P* = 0.083; Rab7: *P* = 0.149; and Rab11: *P* = 0.054). Kaplan-Meier revealed a mean 5-year overall survival rate of 32.2 months (1–120 months; SD ± 8.9). The stratified multivariate survival analysis indicated that co-overexpression of Rab5, Rab7 and Rab11 occurs in advanced stages and correlates with a worst overall survival probability (log-rank test, *P* = 0.006) (Figure 2B). Overexpression of these three Rab members together was considered to possess the best prognostic marker amongst all the possible combinations tested (Figure 2B), suggesting functional cooperation in promoting OSCC progression to metastasis.

**Table 2:** Summary of Rab immunohistochemistry staining in OSCC cases according to demographic, lifestyle, and clinical variables

Variable	Category	Rab5			Rab7			Rab11		
		Negative	Positive	P*	Negative	Positive	P*	Negative	Positive	P*
Age (median)	< 66 year	3 (10.3)	26 (89.7)	0.067	2 (6.9)	27 (93.1)	0.455	12 (41.4)	17 (58.6)	0.642
	≥ 66 year	7 (30.4)	16 (69.6)		3 (13)	20 (87)		11 (47.8)	12 (52.2)	
Gender	Male	5 (16.1)	26 (83.9)	0.490	5 (16.1)	26 (83.9)	0.530	12 (38.7)	19 (61.3)	0.330
	Female	5 (23.8)	16 (76.2)		0	21 (100)		11 (52.4)	10 (47.6)	
Smoking habit	No	6 (26.1)	17 (73.9)	0.264	3 (13)	20 (87)	0.455	10 (43.5)	13 (56.3)	0.922
	Yes	4 (13.8)	25 (86.2)		2 (6.9)	27 (93.1)		13 (44.8)	16 (55.2)	
Alcohol consumption	No	4 (26.7)	11 (73.3)	0.386	0	15 (100)	0.134	8 (53.3)	7 (46.7)	0.400
	Yes	6 (16.2)	31 (83.8)		5 (13.5)	32 (86.5)		15 (40.5)	22 (59.5)	
T stage	T1 + T2	6 (18.8)	26 (81.2)	0.386	**1 (3.1)**	**31 (96.9)**	**0.045**	**16 (50)**	**16 (50)**	**0.049**
	T3 + T4	4 (20)	16 (80)		**4 (20)**	**16 (80)**		**7 (35)**	**13 (65)**	
Lymph nodes	N0	8 (20)	32 (80)	0.797	5 (12.5)	35 (87.5)	0.198	18 (45)	22 (55)	0.838
	N+	2 (16.7)	10 (83.3)		0	12 (100)		5 (41.7)	7 (58.3)	
Recurrence or metastasis	No	**10 (25)**	**30 (75)**	**0.050**	4 (10)	36 (90)	0.864	18 (45)	22 (55)	0.838
	Yes	**0**	**12 (100)**		1 (8.3)	11 (91.7)		5 (41.7)	7 (58.3)	
Status	Alive	8 (17.8)	37 (82.2)	0.500	4 (8.9)	41 (91.1)	0.652	21 (46.7)	24 (53.3)	0.370
	Dead	2 (28.6)	5 (71.4)		1 (14.3)	6 (85.7)		2 (28.6)	5 (71.4)	

Confidence intervals (95%; log-rank test) determined based on normalized mean intensity value units of each Rab determined by quantitative evaluation of IHC

### Rab5, Rab7 and Rab11 expression levels are elevated in OSCC cells and down-regulation of these Rabs inhibited cell migration and invasion

**Figure 3:**
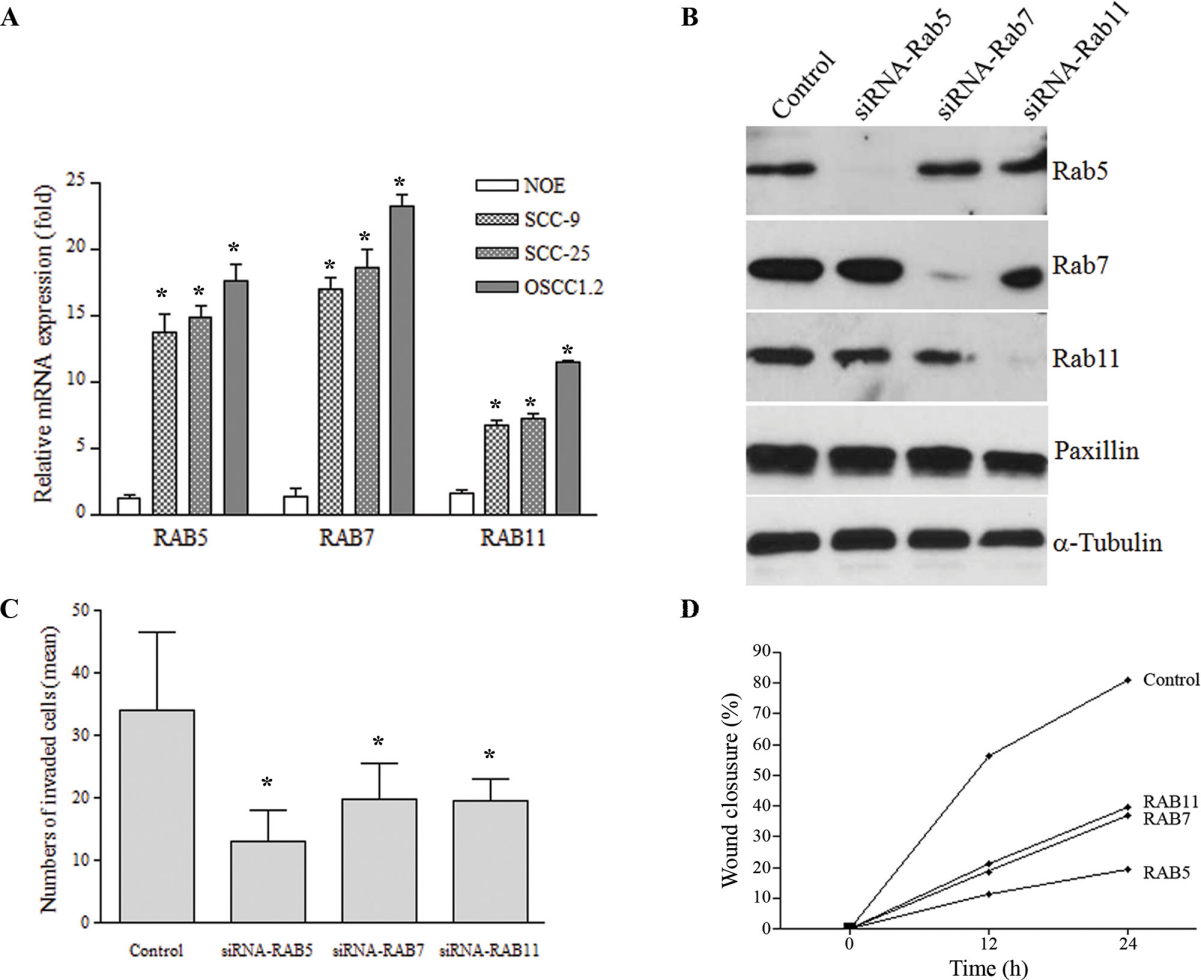
A. mRNA levels of *RAB5*, *RAB7* and *RAB11* estimated by qRT-PCR was elevated in a highly metastatic cell line (OSCC1.2) established from a poorly differentiated and metastatic human oral cancer (stage: T4N2b) with vascular, lymphatic and perineural invasion, as well as in the SCC-25 cell line while low expression was seen in normal oral epithelial (NOE) cells established from normal human tongue tissue (*P < 0.05) GAPDH was used as a reference gene. Y-axis corresponds to the relative quantification of transcript levels and X-axis represents the genes. **B.** Western blot analysis showing efficient down-regulation (> 90%) of endogenous Rab5, Rab7 and Rab11 proteins compared to control cells. *GAPDH* was used as an internal control. **C.** Invasion capacity using the Boyden chamber assay on control OSCC cells and their matched cells expressing siRNA targeting Rab5, Rab7 or Rab11. Bar graph represents the mean number of invaded cells (*P < 0.05). **D.** Cell migration, analyzed by the wound healing assay during 24 h, revealed that OSCC cell migration into the scratched area was inhibited when Rab expression was inhibited by siRNA. Bar graph represents the mean ± SD of five independent experiments, and representative images of the wound captured at different time points are shown.

To investigate the implication of Rab overexpression to OSCC progression, the impact of Rabs on cell invasiveness was monitored in preclinical OSCC models. Firstly, mRNA transcripts and protein levels were quantified for Rab5, Rab7 and Rab11 in a panel of OSCC cell lines: SCC-9, SCC-25 and OSCC1.2; the later cell line was established from a poorly differentiated and metastatic human oral cancer (stage: T4N2b) with vascular, lymphatic and perineural invasion [32, 33]. A non-tumorigenic oral epithelial (NOE) cell line established from normal human tongue tissue [34] was used as control. As shown in Figure 3A, mRNA expression levels of Rab5, Rab7 and Rab11 were elevated in the OSCC cell lines compared to NOE cells (*P* < 0.001). Focusing on SCC-25 cell line, siRNA knockdown of Rab5, Rab7 or Rab11 resulted in a robust down-regulation of endogenous Rab expression (Figure 3B), in comparison to control cells expressing empty plasmid. Moreover, knockdown of these Rab members significantly inhibited cell migration and cell invasion analyzed by Boyden chamber assays (Figure 3C) and wound healing (Figure 3D), respectively (*P* < 0.05). For instance, the wound healing assay revealed that > 88% of the wound was closed within 48 hours by control cells compared to only 11%, 22.6% and 56% in matched *RAB5-*, *RAB7-*, and *RAB11*-silenced cells, respectively.

### Cell locomotion and focal adhesion turnover are regulated by Rab family members

**Figure 4:**
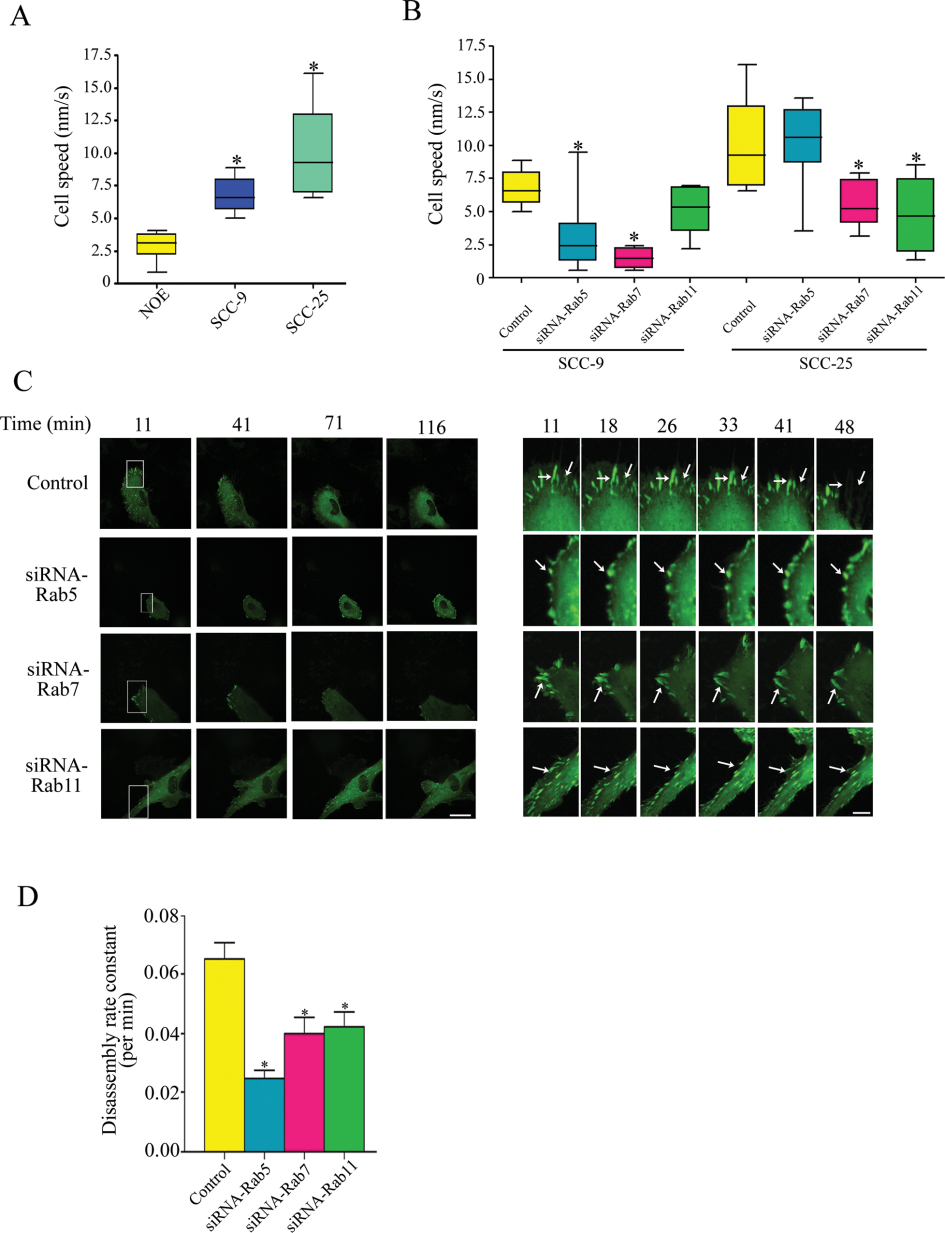
A. Cell motility of NOE, SCC-9 and SCC-25 cells. Cell speed of indicated cells was quantified and plotted as box and whisker plots Cells were traced and measured by Volocity software. Results are plotted as a boxplot where the box spans the 25th and 75th percentile. The average speed was shown in the box and the whisker marks the minimum and maximum data points (*n* = 30 cells per condition); *P < 0.05. **B.** SCC-9 and SCC-25 cells were transfected with the indicated siRNA for Rab5, Rab7 and Rab11, respectively, cell speed was recorded and quantified as (A) (*n* = 30 cells per condition); *P < 0.05. **C.** Control and Rab-siRNA treated OSCC cells were transfected with GFP-paxillin, stimulated with 10 ng/mL EGF, and then analyzed by time-lapse spinning disk confocal microscopy. Representative frames are shown in the left panel, scale bar: 15 μm. Enlargements of boxed regions are shown on the right panel, white arrows indicate focal adhesions (FAs, represented by GFP-paxillin) persistence, scale bar: 5 μm. **D.** Quantification of FAs was measured by counting GFP-paxillin area. Disassembly rates, calculated as described in Material and Method, are presented as means ± SD from at least three independent experiments; Statistical significance was analyzed using the Student's *t* test.*P < 0.05.

The impact of Rab on OSCC cell locomotion was examined *in vitro* using wound-healing assay, as well as single live cell imaging using time-lapse confocal microscopy. The later assay allowed us to rule out a possible contribution of changes in cell proliferation to differences in cell migration when using the wound-healing assay. In agreement with the results from the wound-healing assay, cell motility was observed to be higher in the invasive OSCC cells, while NOE cells were less motile (Figure 4A) (*P* < 0.05). Interestingly, knockdown of Rab5, Rab7 or Rab11 led to a significant inhibition of cell locomotion, as well as protrusive activity (formation of plasma membrane protrusions) (Figure 4B). To further understand the mechanism by which these Rabs regulate cancer cell locomotion, we investigated the impact of Rab on focal adhesion (FA) turnover, a critical mechanism by which cells acquire autonomous motile properties. Control cells and their matched counterparts expressing Rab5-, Rab7- or Rab11-siRNA were transfected with GFP-paxillin (a marker of mature FA) and cultured on fibronectin-coated chamber slides stimulated with 10ng/mL EGF (to promote cell motility and FA formation). In protrusive regions of control cells, the intensity of paxillin-containing adhesions decreased and eventually disappeared as new FA formed at the leading edge (Figure 4C). Rab-silenced cells showed more stable FA structures in contrast to control cells that showed a higher migratory rate and rapid FA disassembly (Figure 4C–4D). Stagnant GFP-paxillin in adhesion complexes was observed in Rab-silenced cells for an extended duration compared to control cells (*P* < 0.01, Figure 4D). Moreover, the rate of GFP-paxillin removal from adhesion sites (quantified by integrating the GFP-paxillin fluorescent intensity in individual adhesions over time) was increased in Rab-silenced cells (*P* < 0.01, Figure 4D). These results document a regulation of FA turnover by Rab5, Rab7 and Rab11 in invasive OSCC cells.

## DISCUSSION

OSCC progression to metastasis is often seen at initial diagnosis or following recurrence. In metastatic OSCC, prognosis and therapeutic outcome remain unpredictable, as most clinically available therapeutics often have limited efficacy and rarely lead to a cure. Search for alternative therapeutic options depends on the molecular understanding of the biology of OSCC metastatic process, which is contributed by factors intrinsic to cancer cells, their microenvironment both at primary sites and distant organs (metastatic niche), as well as host factors.

Here we identified predominant amplifications of chromosomal regions that encompass Rab5, Rab7 and Rab11 GTPases in metastatic compared to non-metastatic OSCC. Rab GTPases play a broad physiological function in vesicular trafficking within a cell and between neighboring cells in the tissue microenvironment [18]. The human genome contains at least 70 Rab members, excluding alternative splice variants that can result in multiple isoforms [37, 38]. In this study, the mRNA and protein expression of Rab5, Rab7 and Rab11 revealed higher levels in invasive compared to non-invasive OSCC supporting their potential use as biomarkers. In particular, Rab5 overexpression was strongly correlated with the occurrence of tumor relapse, while Rab7 and Rab11 overexpression was consistently seen in advanced clinical stages. To determine if Rab members could have a prognostic value, survival probability was compared in cases with negative/low *versus* positive/high Rab expression. A significantly lower survival probability was specifically observed in patients whose tumors co-overexpress Rab5, Rab7 and Rab11. This is not surprising as coordinated regulation of Rab during the multistep intravesicular pathways is established [12, 13]. For instance, Rab5 and Rab7 have been reported to accelerate thyroglobulin endocytosis and subsequent transfer into the proteolytic compartment, and therefore act as tandem regulators of thyroid hormone production in thyroid cancer [38, 39]. In our study, we observed that the expression levels of individual Rab proteins impact the overall survival. However, stratified analyzes revealed that co-overexpression of Rab5, Rab7 and Rab11 has the strongest prognostic values for worst survival rates.

The recurrent amplification mapped on 3p24-p22, 3q21.3 and 8p11–12, corresponding to *RAB5*, *RAB7* and *RAB11*, respectively, support their implication in metastasis progression. Our study identified these Rabs to strongly correlate with poor outcome in OSCC patients and play a role in regulating OSCC cell migration. In head and neck squamous cell carcinoma (HNSCC), one study investigated the expression of Rab-coupling protein (RCP) in 95 heterogeneous HNSCC, 18 vocal nodule epithelia and 16 leukoplakia samples by immunohistochemistry in relation to clinicopathological parameters and outcome. The authors reported that RCP protein contributes to the malignant progression and serves as a prognostic marker in HNSCC patients [8]. Furthermore, two studies demonstrated that Rab25 down-regulation plays an important role in HNSCC progression [14, 40]. However, extrapolation of this observation to tongue OSCC, which is the focus of this study, is premature since oral cavity cancers represented less than 10% of the HNSCC cases investigated in the previous studies addressing Rab25. In our study we cannot exclude the possibility that regional amplifications that target other Rabs partners may be narrow and hence may be missed by aCGH. Noticeable, no difference was seen for RCP between our cohort of metastatic *versus* non-metastatic OSCC cases; however, the chromosomal region of Rab25 was amplified in 50% of our patients whose tumors developed distant metastasis, arguing for an implication of this Rab in OSCC progression and supporting distinct Rab abnormalities in OSCC [41, 42]. Interestingly, studies using animal models showed that mice with genetic deletion of Rab25 develop normally but become more susceptible to carcinogenesis when crossed with heterozygous Smad3+/^_^ mice, clearly demonstrating the requirement for its cooperation with additional oncogenic events [43]. Additional molecular studies are needed to further investigate the cooperation between Rab members, their effectors and established oncogenic events in order to reconcile discrepancies between tumor suppressors *versus* promoter activity of Rab25 in HNSCC.

**Figure 5:**
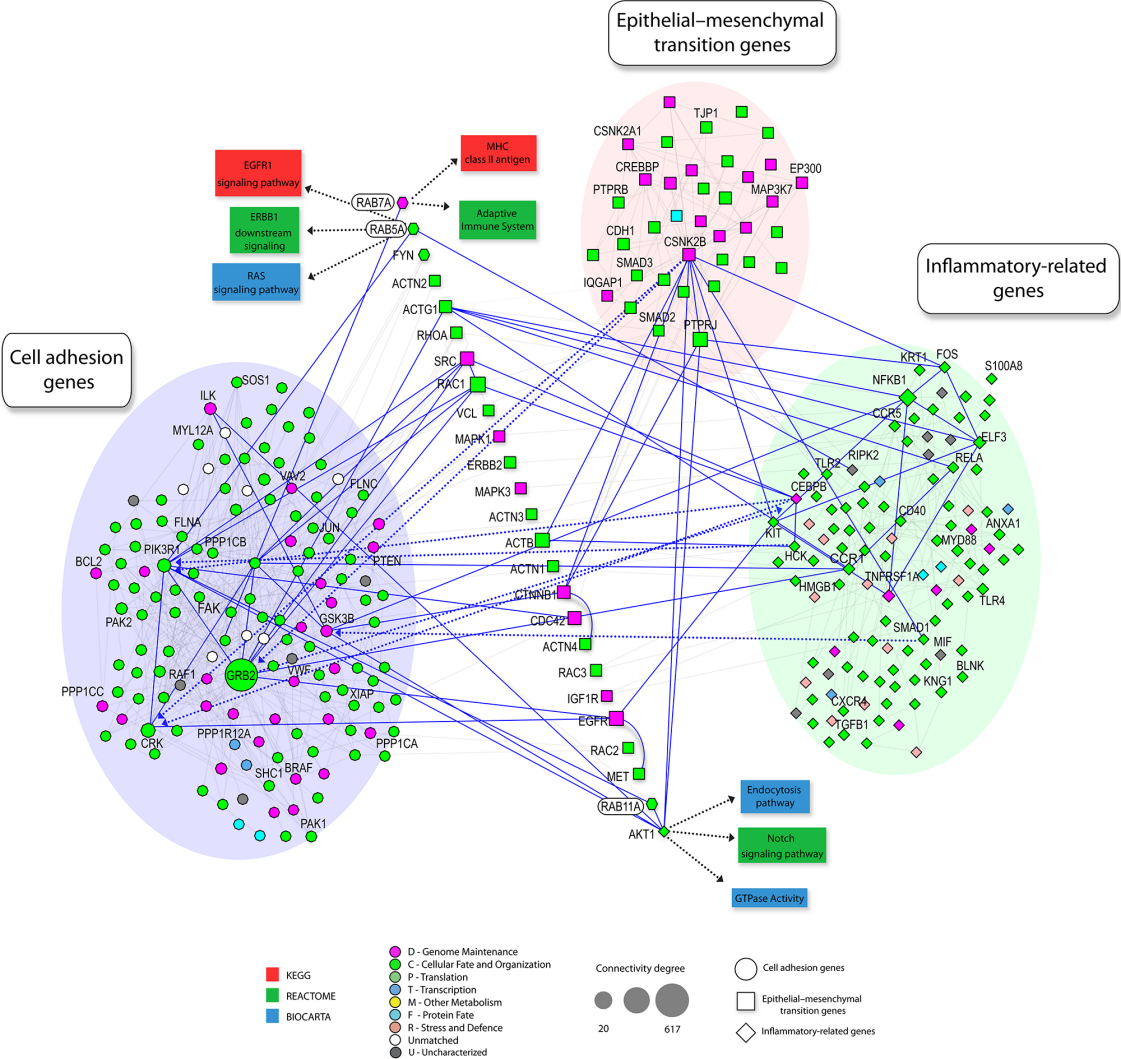
Schematic representation of protein–protein interaction network involving Rab GTPases Shown are protein–protein interaction networks from in NAViGaTOR using I2D and the Reactome, BioCarta and KEGG databases assessing functional interactions. High connectivity (*k* > 20 ) was highlighted by different shapes. GO term enrichment of proteins is rendered by node color. Node shapes indicate the involved process: cell adhesion genes (circle), epithelial-mesenchymal transition genes (square), inflammatory related-genes (diamond). Solid blue edges describe the interactions between well-described enriched genes in each assessed process and neighborhood. Enrichment of proteins involved in cell adhesion genes (blue circle), inflammatory related-genes (green circle) and epithelial-mesenchymal transition genes (pink circle) were observed (*P* < 0.01).

Co-amplification and co-overexpression of Rab5, Rab7 and Rab11 in advanced OSCC supports a functional cooperation. Under physiological conditions, Rab proteins cooperate for protein cycling by regulation of specific steps of protein trafficking. For instance, Rab5 regulates early endocytosis and endosome biogenesis [44], Rab7 is involved in vesicle transport from endosomes to late lysosomes [45] and Rab11 primarily regulates the recycling of protein endocytosis via perinuclear endosomes [46]. To identify potential signaling loops involving the Rab-GTPases to be co-overexpressed in metastatic OSCC, we used a computational approach in which we mined literature-based molecular interaction networks. We further combined initial hub results from centralities and random analyses of the networks with experimental results of our metastatic OSCC cohort to identify potential signaling patterns associated with tumor aggressiveness (Figure 5). Focal adhesion molecules were identified as hubs for invasiveness process related with Rab overexpression. In preclinical OSCC models, our study clearly showed that inhibition of Rab5, Rab7 or Rab11 regulated FA turnover, namely via inhibition of FA disassembly and this in turn prevented cell migration and invasion. This is in accordance with our previous finding showing that rapid FA disassembly is a common mechanism seen in highly invasive cancer cells [35, 47, 48]. FAs have a broad function in cell invasion signaling, in particular they can serve as traction forces for cell migration, as well as scaffolding proteins that mediate multiple protein-protein interactions critical for the early steps of metastasis [49, 50]. Dynamic regulation of FA assembly and disassembly is essential for promoting cell adhesion and motility. FA assembly at the leading edge of the cell facilitates cell anchoring and generation of contractile forces necessary for cell translocation. On the other hand, concomitant FA disassembly at the trailing edge of the cell is required for cell detachment during cell migration [36]. Our results support that Rab GTPases promote accelerated FA turnover in OSCC, a prerequisite for FA disassembly. In summary, our study identified predominant amplifications of chromosomal regions encoding *RAB5*, *RAB7* and *RAB11* genes and supports cooperation among these multiple Rab members for cell invasion. As well, our results highlight the potential utility of pan-Rab inhibitors as a therapeutic approach for invasive OSCC.

## SUPPLEMENTARY TABLES


