# Supplementary material for: Predominant Rab-GTPase amplicons contributing to oral squamous cell carcinoma progression to metastasis
Source: Oncotarget. 2015 Jun 15;6(26):21950–63. doi: 10.18632/oncotarget.4277 (PMC4673138; doi:10.18632/oncotarget.4277)
Supplement: Supplementary file 1 [file oncotarget-06-21950-s001.pdf]

SUPPLEMENTARY TABLES

Supplementary Table S1: PCR primer set sequences and location of the genes selected for qRT-PCR quantification

Gene symbol	Foward primer (5′-3′)	Reverse primer (5′-3′)	Cytogenetic band
RAB5	TTAGAAAAGCAGCCCCAATG	GTACTTCTGGGAGAGTCCGC	3p24-p22
RAB7	CCTTCAGCAACACTTTCTTCCT	CCGTTTAGTCTCCTCCTCGG	3q21.3
RAB11	GCTCGGCCTCGACAAGTTC	ACTTATACCACTGCGTCTTCCT	8p11.22
GAPDH	AATGAAGGGGTCATTGATGG	AAGGTGAAGGTCGGAGTCAA	12p13
ACTB	GCACCCAGCACAATGAAG	CTTGCTGATCCACATCTGC	7p22
HPRT1	GAACGTCTTGCTCGAGATGTGA	TCCAGCAGGTCAGCAAAGAAT	Xq26.1
BCRP	CCTTCGACGTCAATAACAAGGAT	CCTGCGATGGCGTTCAC	4q22

Supplementary Table S2: Identification of genes in frequently gained/amplified chromosome regions of RAB5 (Chromosome 3 - Cytoband p24.3-p11.1) in metastatic oral squamous cell carcinoma

Chromosome 3 Cytoband p24.3-p11.1	Gene Name
AZI2	5-azacytidine induced 2
CMC1	COX assembly mitochondrial protein homolog (S. cerevisiae)
DAZL	deleted in azoospermia-like
DPH3	DPH3, KTI11 homolog (S. cerevisiae); DPH3B, KTI11 homolog B (S. cerevisiae)
EFHB	EF-hand domain family, member B
EOMES	eomesodermin homolog (Xenopus laevis)
GADL1	glutamate decarboxylase-like 1
KAT2B	K(lysine) acetyltransferase 2B
KCNH8	potassium voltage-gated channel, subfamily H (eag-related), member 8
LOC152024	hypothetical LOC152024
LRRC3B	leucine rich repeat containing 3B
NEK10	NIMA (never in mitosis gene a)- related kinase 10
NGLY1	N-glycanase 1
NKIRAS1	NFKB inhibitor interacting Ras-like 1
NR1D2	nuclear receptor subfamily 1, group D, member 2
OXNAD1	oxidoreductase NAD-binding domain containing 1
OXSM	3-oxoacyl-ACP synthase, mitochondrial
PLCL2	phospholipase C-like 2
RAB5A	RAB5A, member RAS oncogene family
RARB	retinoic acid receptor, beta
RBMS3	RNA binding motif, single stranded interacting protein
RFTN1	raftlin, lipid raft linker 1
RPL15	ribosomal protein L15 pseudogene 22; ribosomal protein L15 pseudogene 18; ribosomal protein L15 pseudogene 17; ribosomal protein L15 pseudogene 3; ribosomal protein L15 pseudogene 7; ribosomal protein L15
SATB1	SATB homeobox 1
SGOL1	shugoshin-like 1 (S. pombe)
SLC4A7	solute carrier family 4, sodium bicarbonate cotransporter, member 7
STT3B	STT3, subunit of the oligosaccharyltransferase complex, homolog B (S. cerevisiae)
TBC1D5	TBC1 domain family, member 5
TGFBR2	transforming growth factor, beta receptor II (70/80kDa)
THRB	thyroid hormone receptor, beta (erythroblastic leukemia viral (v-erb-a) oncogene homolog 2, avian)
TOP2B	topoisomerase (DNA) II beta 180kDa
UBE2E1	ubiquitin-conjugating enzyme E2E 1 (UBC4/5 homolog, yeast)
UBE2E2	ubiquitin-conjugating enzyme E2E 2 (UBC4/5 homolog, yeast)
VENTXP7	VENT homeobox (Xenopus laevis) pseudogene 7
ZCWPW2	zinc finger, CW type with PWWP domain 2
ZNF385D	zinc finger protein 385D

Supplementary Table S3: Identification of genes in frequently gained/amplified chromosome regions of RAB7 (Chromosome 3 - Cytoband q11.2-q29) in metastatic oral squamous cell carcinoma

Chromosome 3 Cytoband q11.2-q29	Gene Name
ACAD9	acyl-Coenzyme A dehydrogenase family, member 9
C3orf25	chromosome 3 open reading frame 25
C3orf27	chromosome 3 open reading frame 27
C3orf37	chromosome 3 open reading frame 37
C3orf47	chromosome 3 open reading frame 47
CCDC48	coiled-coil domain containing 48
CNBP	CCHC-type zinc finger, nucleic acid binding protein
COPG	coatomer protein complex, subunit gamma
DNAJB8	DnaJ (Hsp40) homolog, subfamily B, member 8
GATA2	GATA binding protein 2
GP9	glycoprotein IX (platelet)
H1FOO	H1 histone family, member O, oocyte-specific
H1FX	H1 histone family, member X
IFT122	intraflagellar transport 122 homolog (Chlamydomonas)
ISY1	ISY1 splicing factor homolog (S. cerevisiae)
KIAA1257	KIAA1257
LOC90246	hypothetical protein LOC90246
MBD4	methyl-CpG binding domain protein 4
PLXND1	plexin D1
RAB43	RAB43, member RAS oncogene family; hypothetical LOC100131426
RAB7A	RAB7A, member RAS oncogene family
RHO	rhodopsin
RPL32P3	ribosomal protein L32 pseudogene 3
RPN1	ribophorin I
SNORA7B	small nucleolar RNA, H/ACA box 7A; small nucleolar RNA, H/ACA box 7B; ribosomal protein L32

Supplementary Table S4: Identification of genes in frequently gained/amplified chromosome regions of RAB11(Chromosome 8 - Cytoband p23.3-p11.1) in metastatic oral squamous cell carcinoma

Chromosome 8 Cytoband p23.3-p11.1	Gene Name
ADRB3	adrenergic, beta-3-, receptor
ASH2L	ash2 (absent, small, or homeotic)-like (Drosophila)
BAG4	BCL2-associated athanogene 4
BRF2	BRF2, subunit of RNA polymerase III transcription initiation factor, BRF1-like
C8orf86	chromosome 8 open reading frame 86
DDHD2	DDHD domain containing 2
EIF4EBP1	eukaryotic translation initiation factor 4E binding protein 1
ERLIN2	ER lipid raft associated 2
FGFR1	fibroblast growth factor receptor 1
GOT1L1	glutamic-oxaloacetic transaminase 1-like 1
GPR124	G protein-coupled receptor 124
LETM2	leucine zipper-EF-hand containing transmembrane protein 2
LOC728024	hCG1640171
LSM1	LSM1 homolog, U6 small nuclear RNA associated (S. cerevisiae)
PPAPDC1B	phosphatidic acid phosphatase type 2 domain containing 1B
PROSC	proline synthetase co-transcribed homolog (bacterial)
RAB11	RAB11 family interacting protein 1 (class I)
RNF5P1	ring finger protein 5; ring finger protein 5 pseudogene 1
STAR	steroidogenic acute regulatory protein
WHSC1L1	Wolf-Hirschhorn syndrome candidate 1-like 1

Supplementary Table S5: Cross-referenced of the 72-RAB cluster genes with list of differentially expressed genes in Oral Squamous Cell Carcinoma, retrieved from the data collected and processed at the NCBI-GEO database (http://www.ncbi.nlm.nih.gov/geo/) originated from ten studies reported in the literature. It was considered a $p < 0.05$ as significant. Protein type was classified as GEFs (guanine nucleotide exchange factors), GAPs (GTPase activating proteins) and effector proteins

RAB central	Gene partner	Gene Name	Protein type	[1]Sheu et al. 2013	[2]Sumino et al. 2013	[3]Reis et al. 2011	[4]Chen et al. 2008	[5]Ambatipudi et al. 2012	[6]Estilo et al. 2011	[7]Sticht et al. 2008	[8]Ye et al. 2008	[9]Toruner et al. 2004	[10]O'Donnell et al. 2005
RAB11A	*GNB1*	Guanine nucleotide binding protein (G protein)	Eff	−0.09		0.06	0.14	12.11	0.81	−0.08	0.24	0.42	
RAB11A	*PIK3R1*	Phosphoinositide-3-kinase, regulatory subunit 1	GAP	0.89		−0.27	−0.05	8.57	−1.08	−0.48		0.48	
RAB11A	*MYO5A*	Myosin VA	Eff	−0.53		0.22	0.55	19.57	2.41		0.28	0.65	
RAB11A	*RAB11FIP1*	RAB11 family interacting protein 1	Eff	0.63		−0.25	−0.14	−11.42		−0.40	−0.15	−1.00	
RAB5A	*ALS2CL*	ALS2 C-terminal like	GEF	−0.86		−0.41	−0.31	−7.04		81.66	−0.17	−0.86	
RAB5A	*RIN1*	Ras and Rab interactor 1	GEF	−0.91			0.05	9.01	1.03	0.12	0.03	−0.69	
RAB5A	*RIN2*	Ras and Rab interactor 2	GEF	−0.13		0.08	0.14		1.37	−7.00	0.16	1.43	
RAB5A	*CAV1*	Caveolin 1, caveolae protein	Eff	−0.32		0.11	0.29	−37.92	1.09	−0.39	0.12		
RAB5A	*PIK3R4*	Phosphoinositide-3-kinase, regulatory subunit 4	Eff	−0.67		0.07	0.08		0.50	−0.14	0.04	1.01	
RAB7A	*OSBPL1A*	Oxysterol binding protein-like 1A	Eff	0.45		−0.13	−0.12	5.19	−0.98	−0.23	−0.12		
RAB7A	*PSMA7*	Proteasome (prosome, macropain) subunit, alpha type, 7	Eff			0.07	0.06	27.87	−0.38	−0.60	0.20	0.42	
RAB11A	*ARHGAP1*	Rho gtpase activating protein 1	Eff	−0.23		0.04	0.07		0.76	−1.15	−0.06		
RAB11A	*MYO5B*	Myosin VB	Eff	0.82		−0.31	−0.31	11.80		0.40	−0.11		
RAB11A	*RAB11FIP5*	RAB11 family interacting protein 5	Eff	−0.39		0.03	0.10	−23.07		0.08	0.04		
RAB11A	*TRPV6*	Ransient receptor potential cation channel, subfamily V, member 6	Eff	3.40		−0.05	−0.05	102.14	−1.11		−0.13		
RAB5A	*LRRK2*	Leucine-rich repeat kinase 2	Eff	1.64		−0.41	−0.45	−17.80		0.20	−0.15		
RAB5A	*RUFY1*	RUN and FYVE domain containing 1	Eff	0.23		−0.08	−0.09	−15.76		0.24	−0.10		
RAB5A	*SDC1*	Syndecan 1	Eff	−0.26			−0.06		1.18	−1.15	0.11	−1.32	
RAB5A	*ZFYVE20*	Zinc finger, FYVE domain containing 20	Eff	0.20		−0.01	−0.03	4.69		0.29	−0.06		
RAB7A	*FYCO1*	FYVE and coiled-coil domain containing 1	Eff	1.21		−0.21	−0.15	−8.71		−0.10	−0.18		
RAB7A	*VPS26A*	Vacuolar protein sorting 26	Eff	0.16		−0.03	−0.04	56.49	0.54	0.19			
RAB11A	*AKAP10*	A kinase (PRKA) anchor protein 10	Eff			0.24	0.11		0.51	−0.20	−0.21		
RAB11A	*PSEN1*	Presenilin 1	Eff	−0.41		0.06			0.40	0.00	0.08		
RAB11A	*PSEN2*	Presenilin 2	Eff	0.17			−0.14		−0.83	−0.52	−0.01		

(*Continued*)

RAB central	Gene partner	Gene Name	Protein type	[1]Sheu et al. 2013	[2]Sumino et al. 2013	[3]Reis et al. 2011	[4]Chen et al. 2008	[5]Ambatipudi et al. 2012	[6]Estilo et al. 2011	[7]Sticht et al. 2008	[8]Ye et al. 2008	[9]Toruner et al. 2004	[10]O'Donnell et al. 2005
RAB11A	*RAB11FIP2*	RAB11 family interacting protein 2	Eff	0.22		−0.03	−0.05		−0.51	−0.46			
RAB11A	*RAB3IP*	RAB3A interacting protein	Eff	−0.11			0.11	−391.29		−0.07	0.18		
RAB11A	*SMCHD1*	Structural maintenance of chromosomes flexible hinge domain containing 1	Eff			0.07	0.10			0.19	0.08	0.94	
RAB11A	*TBXA2R*	Thromboxane A2 receptor	Eff	−0.06	−0.57		0.01			−0.03		−0.58	
RAB5A	*ALS2*	Amyotrophic lateral sclerosis 2	GEF	0.09			0.09	−14.23		0.04	0.09		
RAB5A	*GAPVD1*	Gtpase activating protein and VPS9 domains 1	GEF	0.15			−0.06		0.59		−0.05	−0.71	
RAB5A	*TSC2*	Tuberous sclerosis 2	GAP	−0.75				−4.99		−0.11	0.05	1.11	
RAB5A	*USP6NL*	USP6 N-terminal like	GAP	0.48		−0.21	−0.36	−72.45			−0.23		
RAB5A	*AGTR1*	Angiotensin II receptor, type 1	Eff	2.90		−0.33		−8.79	−2.91	0.16			
RAB5A	*EEA1*	Early endosome antigen 1	Eff			−0.12	−0.10	−5.95	−0.58		−0.12		
RAB5A	*PIK3C3*	Phosphatidylinositol 3-kinase, catalytic subunit type 3	Eff	0.09			−0.03	110.05		−0.05	−0.13		
RAB5A	*RABAC1*	Rab acceptor 1 (prenylated)	Eff	−0.39		0.10	0.09			−0.26	0.05		
RAB5A	*RABEP2*	Rabaptin, RAB gtpase binding effector protein 2	Eff	−0.36			−0.09	11.11		−0.09	−0.03		
RAB5A	*SDCBP*	Syndecan binding protein (syntenin)	Eff	−0.16			0.04	9.28		−0.07		0.75	
RAB5A	*VPS11*	Vacuolar protein sorting 11	Eff	0.18		−0.08	−0.12			−0.17	−0.09		
RAB7A	*VPS29*	Vacuolar protein sorting 29	Eff	0.08	0.37	−0.02	0.08			−0.49			
RAB11A	*GNG2*	Guanine nucleotide binding protein	Eff	0.38			0.01	5.19			−0.11		
RAB11A	*PI4KB*	Phosphatidylinositol 4-kinase	Eff				−0.01	−33.64			0.04	−0.40	
RAB5A	*RIN3*	Ras and Rab interactor 3	GEF	−1.08			0.04			−0.30		0.49	
RAB5A	*RASA1*	RAS p21 protein activator	GAP	−1.39			−0.02		0.61		0.07		
RAB5A	*APPL1*	Adaptor protein, phosphotyrosine interaction	Eff			−0.22	−0.18	−94.38			−0.23		
RAB5A	*OCRL*	Oculocerebrorenal syndrome of Lowe	Eff	0.11			0.03		−0.42			−0.20	
RAB5A	*PIK3CB*	Phosphatidylinositol-4,5-bisphosphate 3-kinase	Eff	0.13			−0.08		−0.44	−0.17			
RAB5A	*RABEP1*	Rabaptin, RAB gtpase binding effector protein 1	Eff				0.13	−45.61	−0.33		0.06		
RAB7A	*RAC1*	Ras-related C3 botulinum toxin substrate 1	Eff	−0.52			−0.02		0.74	35.84			

(*Continued*)

RAB central	Gene partner	Gene Name	Protein type	[1]Sheu et al. 2013	[2]Sumino et al. 2013	[3]Reis et al. 2011	[4]Chen et al. 2008	[5]Ambatipudi et al. 2012	[6]Estilo et al. 2011	[7]Sticht et al. 2008	[8]Ye et al. 2008	[9]Toruner et al. 2004	[10]O'Donnell et al. 2005
RAB7A	*RILP*	Rab interacting lysosomal protein	Eff	0.14		−0.09	−0.07	6.30					
RAB11A	*ADRB2*	Adrenoceptor beta 2, surface	Eff	0.11		−0.25				−4.25			
RAB11A	*HOOK1*	Hook homolog 1	Eff				−0.06	38.26				−1.49	
RAB11A	*PTGIR*	Prostaglandin I2 receptor (IP)	Eff	−1.63			0.01			0.01			
RAB11A	*RAB11FIP3*	RAB11 family interacting protein 3	Eff				0.07			0.21			0.23
RAB5A	*RABGEF1*	RAB guanine nucleotide exchange factor 1	GEF	0.18				10.34		−0.04			
RAB5A	*ANKFY1*	Ankyrin repeat and FYVE domain containing 1	Eff	−0.20			0.05			−0.34			
RAB5A	*INPP5B*	Inositol polyphosphate-5-phosphatase	Eff				−0.01		−0.44		−0.06		
RAB5A	*MON1A*	MON1 homolog A	Eff			−0.13		6.40		−0.05			
RAB5A	*NAE1*	NEDD8 activating enzyme E1 subunit 1	Eff					17.91	0.33			0.85	
RAB5A	*SUN2*	Sad1 and UNC84 domain containing 2	Eff				−0.11		−0.39		−0.09		
RAB7A	*VPS39*	Vacuolar protein sorting 39 homolog	GEF		−0.53				−0.57	0.19			
RAB7A	*RNF115*	Ring finger protein 115	Eff				0.05		−0.61			0.64	
RAB7A	*VPS35*	Vacuolar protein sorting 35	Eff				−0.03	−3.77		−0.18			
RAB11A	*TBC1D15*	TBC1 domain family, member 15	GAP	0.04			0.03						
RAB11A	*DENND5A*	DENN/MADD domain containing 5A	Eff				0.11	7.95					
RAB11A	*EXOC6*	Exocyst complex component 6	Eff	0.68						0.07			
RAB11A	*VPS33B*	Vacuolar protein sorting 33	Eff	−0.09			−0.08						
RAB11A	*ZFYVE27*	Zinc finger, FYVE domain containing 27	Eff				0.05			0.27			
RAB5A	*TBC1D17*	TBC1 domain family, member 17	Eff	−0.27				−14.34					
RAB7A	*CCZ1*	Vacuolar protein trafficking and biogenesis associated	Eff					14.10				−0.44	
RAB7A	*PCBP1*	Poly(rc) binding protein 1	Eff				−0.03		−0.38				

[1]Oncogene (2013)
[2]Int J Cancer(2013)132(3):540–8
[3]BMC Cancer (2011)11:437
[4]Cancer Epidemiol Biomarkers Prev (2008)17(8):2152–62
[5]Genes Chromosomes Cancer (2012)51(2):161–73
[6]BMC Cancer (2009)12–9:11
[7]Neoplasia(2008)10(5):462–70
[8]BMC Genomics(2008)6–9:69
[9]Cancer Genet Cytogenet(2004) 154(1):27–35
[10]Oncogene (2005)10–24(7):1244–51

Supplementary Table S6: Cross-referenced of the 61-RAB interacting protein obtained from protein-protein interaction databases: MINT (*mint.bio.uniroma2.it/mint/*), HPRD (*www.hprd.org) and* IntAct (*www.ebi.ac.uk/intact/*). The list of differentially expressed genes in Oral Squamous Cell Carcinoma was collected and processed at the NCBI-GEO database (http://www.ncbi.nlm.nih.gov/geo/) originated from ten studies reported in the literature. It was considered a $p < 0.05$ as significant

RABcentral	RABs PPI Partner	Gene Name	[1]Sheu et al. 2013	[2]Sumino et al. 2013	[3]Reis et al. 2011	[4]Chen et al. 2008	[5]Ambatipudi et al. 2012	[6]Estilo et al. 2011	[7]Sticht et al. 2008	[8]Ye et al. 2008	[9]Toruner et al. 2004	[10]O'Donnell et al. 2005
RAB5A	*ALS2CL*	ALS2 C-terminal like	−0.86		−0.41	−0.42	−7.04		0.07	−0.31	−0.86	
RAB11A	*FTSJ1*	Ftsj RNA methyltransferase homolog 1	−0.16		0.07	0.10		0.54	0.10	0.10		−0.40
RAB5A	*PIK3R1*	Phosphoinositide-3-kinase, regulatory subunit 1	0.65		−0.27	−0.11	8.57	−1.08	−14.64		0.48	
RAB7A	*ATG5*	Autophagy related 5			0.05	0.05	12.69	−0.91	−0.08	0.11	0.70	
RAB5A	*RIN2*	Ras and Rab interactor 2	−0.13		0.08	0.14		1.37	−7.00	0.16	1.43	
RAB11A	*MYO5A*	Myosin VA	−0.53		0.22	0.55	19.57	2.41		0.28	0.65	
RAB5A	*ITGB1*	Integrin, beta 1	−0.25			0.12	−81.80	0.92	−0.18	0.18		
RAB5A	*RAB5C*	**RAB5C**, member RAS oncogene family	−0.60		0.12	0.12	155.61	0.41	−0.11			
RAB7A	*CHM*	Choroideremia (Rab escort protein 1)	−0.27		0.06	0.19	4.27	−0.61		0.02		
RAB11A	*RAB11FIP1*	RAB11 family interacting protein 1 (class I)	0.37		−0.25	−0.30	−11.42		−0.40		−1.00	
RAB5A	*RIN1*	Ras and Rab interactor 1	−0.91			0.05	9.01	1.03	0.12		−0.69	
RAB11A	*PPP2R1B*	Protein phosphatase 2, regulatory subunit A, beta	0.57			−0.04		0.84	−0.09			0.37
RAB7A	*HLA-B*	Major histocompatibility complex, class I, B			0.09	0.13	−7.88		−0.58		2.19	
RAB5A	*SDCBP*	Syndecan binding protein (syntenin)	−0.16			0.04	9.28		−0.07		0.75	
RAB11A	*RAB11FIP2*	RAB11 family interacting protein 2 (class I)	0.22		−0.03	−0.05		−0.51	−0.46			
RAB5A	*CHML*	Choroideremia-like (Rab escort protein 2)			0.24	0.10	10.70		0.35			0.65
RAB7A	*GABARAPL2*	GABA(A) receptor-associated protein-like 2	0.29			−0.04	−44.30	−0.36			0.43	
RAB5A	*USP6NL*	USP6 N-terminal like	0.48		−0.21	−0.74	−72.45			−0.23		
RAB7A	*EIF1B*	Eukaryotic translation initiation factor 1B	0.17		−0.06		4.77	−0.36	−0.31			
RAB5A	*ALS2*	Amyotrophic lateral sclerosis 2 (juvenile)	0.09			0.09	−14.23		0.04	0.09		
RAB11A	*GDI2*	GDP dissociation inhibitor 2	0.05	0.29		−0.02		0.56	−0.16			
RAB11A	*TBXA2R*	Thromboxane A2 receptor	−0.89	−0.57		−0.02			−0.03		−0.58	
RAB7A	*MPG*	N-methylpurine-DNA glycosylase	−0.62	0.30		0.04	66.04		0.07			
RAB5A	*ZFYVE20*	Zinc finger, FYVE domain containing 20	0.20		−0.01	−0.12	4.69		0.14			
RAB11A	*RAB11FIP5*	RAB11 family interacting protein 5 (class I)	−0.39		0.03	0.10	−23.07		0.08			
RAB11A	*MYO5B*	Myosin VB	0.82		−0.31	−0.46	11.80		−2.42			
RAB5A	*AGTR1*	Angiotensin II receptor, type 1	2.90		−0.33		−8.79	−2.91	0.16			

(*Continued*)

RABcentral	RABs PPI Partner	Gene Name	[1]Sheu et al. 2013	[2]Sumino et al. 2013	[3]Reis et al. 2011	[4]Chen et al. 2008	[5]Ambatipudi et al. 2012	[6]Estilo et al. 2011	[7]Sticht et al. 2008	[8]Ye et al. 2008	[9]Toruner et al. 2004	[10]O'Donnell et al. 2005
RAB5A	*OCRL*	Oculocerebrorenal syndrome of Lowe	0.11			0.03		−0.42			−0.20	
RAB11A	*PPP2R1A*	Protein phosphatase 2, regulatory subunit A, alpha		0.32		−0.04		0.42	−0.18			
RAB7A	*RILP*	Rab interacting lysosomal protein	0.14		−0.09	−0.07	6.30					
RAB5A	*TSC2*	Tuberous sclerosis 2	−0.75				−4.99		−0.11		1.11	
RAB5A	*RAB37*	**RAB37**, member RAS oncogene family	0.90		0.00		15.14		−0.19			
RAB11A	*TRAF6*	TNF receptor-associated factor 6	0.19			−0.08	−7.91		0.27			
RAB5A	*EEA1*	Early endosome antigen 1			−0.12	−0.11	−5.95	−0.58				
RAB11A	*PSEN2*	Presenilin 2 (Alzheimer disease 4)	0.17			−0.14		−0.83	−0.52			
RAB5A	*RABAC1*	Rab acceptor 1 (prenylated)	−0.39		0.10	0.09			−0.26			
RAB5A	*TM9SF4*	Transmembrane 9 superfamily protein member 4			0.10	0.09		0.62	0.05			
RAB11A	*ME2*	Malic enzyme 2, NAD(+)-dependent, mitochondrial				0.08	12.98		0.06		1.27	
RAB7A	*GABARAPL1*	GABA(A) receptor-associated protein like 1	0.30					−1.23	−0.24		1.54	
RAB5A	*GDI1*	GDP dissociation inhibitor 1	−0.36		0.08	0.10	−3.02					
RAB11A	*VPS24*	(CHMP3)charged multivesicular body protein 3	0.12		−0.04	−0.10			−1.58			
RAB11A	*STX4*	Syntaxin 4			0.06	0.06	−1.82	0.31				
RAB11A	*RABGGTB*	Rab geranylgeranyltransferase, beta subunit	0.40		−0.03	−0.03	−17.75					
RAB11A	*PSEN1*	Presenilin 1	−0.59		0.06			0.40	−0.35			
RAB7A	*DNM2*	Dynamin 2	−0.29			−0.06			0.08			
RAB11A	*VHL*	Von Hippel-Lindau tumor suppressor	−0.32					−0.62	−0.02			
RAB11A	*CHMP1B*	Charged multivesicular body protein 1B			0.07	0.04	−18.39					
RAB5A	*VPS45*	Vacuolar protein sorting 45 homolog				−0.03		−0.63			0.78	
RAB7A	*RNF115*	Ring finger protein 115				−0.01		−0.61			0.64	
RAB5A	*RABEP1*	Rabaptin, RAB gtpase binding effector protein 1				0.10	−45.61	−0.33				
RAB7A	*ATG9A*	Autophagy related 9A	−0.86					−0.26	−0.04			
RAB7A	*SH3KBP1*	SH3-domain kinase binding protein 1	−0.19			0.22			0.08			
RAB5A	*APPL1*	Adaptor protein, phosphotyrosine interaction			−0.22	−0.22	−94.38					
RAB11A	*DTNBP1*	Dystrobrevin binding protein 1	−0.29			0.09			−0.08			
RAB5A	*RASA1*	RAS p21 protein activator	−1.39			−0.02		0.61				
RAB11A	*RAB11FIP3*	RAB11 family interacting protein 3				0.06			−0.06			0.23
RAB5A	*VPS35*	Vacuolar protein sorting 35				−0.05	−3.77		−0.18			
RAB7A	*SLC2A4*	Solute carrier family 2	0.15				36.28	−1.83				

(*Continued*)

RABcentral	RABs PPI Partner	Gene Name	[1]Sheu et al. 2013	[2]Sumino et al. 2013	[3]Reis et al. 2011	[4]Chen et al. 2008	[5]Ambatipudi et al. 2012	[6]Estilo et al. 2011	[7]Sticht et al. 2008	[8]Ye et al. 2008	[9]Toruner et al. 2004	[10]O'Donnell et al. 2005
RAB7A	*MCC*	Mutated in colorectal cancers	−0.21			0.17						
RAB11A	*ZFYVE27*	Zinc finger, FYVE domain containing 27				0.05			0.10			
RAB7A	*PRKAB1*	Protein kinase, AMP-activated, beta 1 non-catalytic subunit				−0.15			−0.09			

[1]Oncogene (2013)
[2]Int J Cancer(2013)132(3):540–8
[3]BMC Cancer (2011)11:437
[4]Cancer Epidemiol Biomarkers Prev (2008)17(8):2152–62
[5]Genes Chromosomes Cancer (2012)51(2):161–73
[6]BMC Cancer (2009)12–9:11
[7]Neoplasia(2008)10(5):462–70
[8]BMC Genomics(2008)6–9:69
[9]Cancer Genet Cytogenet(2004) 154(1):27–35
[10]Oncogene (2005)10–24(7):1244–51
